# Using journey mapping to understand the patient experience with selecting a Medicare part D plan using a pharmacy consultation service

**DOI:** 10.1016/j.rcsop.2021.100006

**Published:** 2021-03-31

**Authors:** Logan T. Murry, Arwa Al-Khatib, Matthew J. Witry

**Affiliations:** The University of Iowa College of Pharmacy, Department of Pharmacy Practice, Health Services Research, 180 S Grand Avenue, Iowa City, IA 52242, United States of America

**Keywords:** Patient experience, Journey mapping, Service design, Medicare Part D, Community pharmacy services

## Abstract

**Background:**

Patient experience with community pharmacy services can be informed by human-centered design principles and approaches. Pharmacy services may benefit from detailed evaluations of consumer experience and patient-centered service design.

**Objectives:**

To use an online journey mapping platform to understand the patient experience with selecting a Medicare Part D plan for individuals that did, and did not, use a free, pharmacy-led, Medicare Part D consultation service.

**Methods:**

This was a two-group cross-sectional survey study in a single, rural community pharmacy. Surveys consisted of 7 demographic items, 30 Likert-type items, and 7 open-ended response items. The pharmacy used purposeful convenience sampling to distribute a paper survey to individuals 65 years of age and older currently enrolled in Medicare Part D between June and August 2019. Surveys were distributed to 36 patients currently enrolled in a Medicare Part D plan, with 18 surveys distributed to patients who had previously used a pharmacy-led Medicare Part D consultation service and 18 surveys distributed to patients who did not use the service. Surveys were uploaded to an online journey mapping platform, producing data visualizations for each group. Multiple choice survey items were analyzed using descriptive statistics, wth service user and nonuser groups compared using Mann-Whitney *U* tests. Open-ended survey responses were coded by the research team using an inductive approach.

**Results:**

In total, 36 surveys were returned to the community pharmacy for a response rate of 100%. The journey map platform generated *Persona*, *Empathy*, and *Current Journey* outputs, which mapped *Good Experiences* and *Bad Experiences* within the Medicare Part D plan selection experience. *Personas* differed in their median household incomes ($25,000–$39,999 for service users compared to $50,000–$74,999 for nonusers). *Empathy* and *Current Journey* outputs showed that service users had a wider variety of emotions compared to non-users. Mann-Whitney *U* tests yielded 5 items with statistically significant differences (*p*-values <0.05) in the plan-selection experience, with both groups similarly uncertain about their plan decision. Qualitative responses indicate patient trust was universally important to a complex decision-making process.

**Conclusions:**

An online journey mapping platform provided insight into how patients experience a pharmacy service that extends beyond satisfaction. For community pharmacies providing Medicare Part D plan consultation services, pharmacies should consider how they can improve the service experience through communication style and patient-centered service design.

## Highlights

• Design Think practices like journey mapping may aid in patient-centered service design.
• The elaboration likelihood model (ELM) may help to explain patient experience with a Medicare Part D consultation service with Medicare
• Information processing ability and heuristics may influence patient decisions.

## 1 Introduction

The Medicare Part D insurance program began in 2006, providing eligible beneficiaries, including those 65 years of age and older, with outpatient prescription drug coverage.^1^ The Medicare Part D plan selection process may be challenging, due to the large number of plans.^2^ Medicare Part D plans often have varying benefit designs (premiums, copayments, etc.) which may be difficult to understand and further add to beneficiary confusion.^3^ Despite potential out-of-pocket (OOP) cost savings, many beneficiaries do not switch plans during the annual open-enrollment period.^4^, ^5^, ^6^

Pharmacy-led Medicare Part D consultation services have been shown to increase Medicare Part D plan-switching behavior, patient OOP cost-savings, and chronic medication adherence.^6^, ^7^, ^8^, ^9^ Focusing on explanations of medical or drug benefits, Medicare Part D plan comparison, and screening patients for plan eligibility, pharmacy-led Medicare Part D consultation services may be an effective way of improving the beneficiary experience with selecting a Medicare Part D plan.^10^ Despite potential benefits and the increasing availability of pharmacy-led Medicare Part D consultation services, it appears that few patients repeatedly use these services despite positive service experiences and high levels of satisfaction.^11^ Further, a recent study identified that only 2.8% of patients using a pharmacy-led Medicare Part D consultation service in successive years a repeatedlyswitched plans.^9^ While most people who use Medicare Part D plan selection services report positive service experiences and plan-switching intent, few actually switch plans. Previous studies of pharmacy-led Medicare Part D consultation services have been limited by their focus on single-item satisfaction measures to evaluate patient experience.^9^, ^10^, 11. There is a need for more thorough evaluations of the patient experience with pharmacy services to better understand the nuances and dynamic nature of the Medicare Part D plan-selection process.

While satisfaction and willingness-to-pay measures have historically been used to evaluatepatient experience and perceptions of service value, these summative measures can be difficult to interpret and can yield results that are limited in their pragmatic applications to patient-centered service improvement.^12^, ^13^, ^14^ Journey mapping, a practice based on the tenets of Design Thinking, is a methodology used in consumer experience research to generate insights into the patient experience with healthcare service use.^15^ A journey map is a visual representation of the emotions and perceptions an individual may experience throughout a challenging or complex experience.16., 17., 18. Patient-centered journey maps have been used in an institutional healthcare setting with the goal of improving the way inpatient care teams deliver services and educate patients on medications, follow-up care, and provide explanations about problems or conditions.^19^, ^20^, 21., 22 The experiences encountered by an individual are typically mapped out for a specific scenario starting with the initial discovery of a challenge and proceeding through information gathering, analysis of various choices, a subsequent decision or purchase, and follow-on experiences. Qualitative research and anecdotal data are commonly used in creating a journey map, with data collected directly from patients or consumers. In some instances, quantitative research findings can also be incorporated to provide additional support to experience findings.

The hypothesis tested in this study is that existing pharmacy-led Medicare Part D consultation services may not consistently improve the Medicare Part D plan-selection experience. Previous studies have identified that while patients frequently report positive experiences and high levels of satisfaction with community pharmacy Medicare Part D consultation services, they inconsistently use the information gathered from consultation services to switch Part D plans and infrequently use the services in successive years despite potential cost-savings.^9^, ^10^, 11. Further, there is reasonable evidence to inform the hypothesis that patient experience methodology like journey mapping will provide additional insight on the patient experience with a pharmacy-led Medicare Part D consultation service and the Medicare Part D plan-selection experience, as journey maps account for how emotions are felt throughout the patient experience with a challenging decision-making process, rather than the cross-sectional focus of conventional satisfaction assessments.16., 17., 18.

The objective of this study was to use a journey mapping methodology to depict and analyze patient experiences with selecting a Medicare Part D plan for two groups: pharmacy patients who used a pharmacy Medicare Part D consultation service and those who did not. This study makes an important contribution to the literature, using an innovative methodology to assess the patient experience with a community pharmacy service designed to assist patients with a challenging Medicare Part D plan-selection decision.

## 2 Methods

### 2.1 Study design

This was an exploratory study using a two-group cross-sectional paper survey combined with a third-party online journey mapping analysis platform. Surveys were administered between 6 and 8 months after participation in the pharmacy-led consultation service. The study was reviewed and approved by The University of Iowa The Institutional Review Board.

### 2.2 Description of the pharmacy-led medicare part D consultation service

The study pharmacy is independently owned and located in a small rural town in the Midwest United States. The pharmacy offers various services like vaccinations, medication synchronization, and medication therapy management (MTM). Medicare Part D consultations are available at the community pharmacy year-round for individuals 65 years of age and older or for those looking to enroll in a Medicare Part D plan for the first time. The pharmacy provided information and resources to be used for Medicare Part D plan-selection decisions during the open enrollment period: October 15th to December 7th, 2018 All consultations were provided by one of two individuals: the supervising pharmacist or one certified technician with Medicare Part D plan-selection experience and training. Data on who provided the consultation could not be linked to the individual survey response.

Patients eligible for Medicare Part D were mailed a letter in early October 2018, notifying them of the upcoming open-enrollment period and offering free Medicare Part D consultations. For interested parties, consultations were scheduled in 30-min blocks at the community pharmacy. If patients were unable or unwilling to receive the consultation in-person, the same information was offered via a telephone consultation. Before the scheduled consultation, the pharmacist completed a comprehensive medication review (CMR), with third-party software (Amplicare, FDS INC. 2020) used to compare Medicare Part D plans based on the current medication regimen. The pharmacy provided each patient with a statement disclosing that the consultations were conducted with the patient's best interests in mind.

The pharmacy staff member conducting the service verified the patient's current Medicare Part D plan before printing off the plan-comparison output generated by pharmacy software. This software evaluated patients' chronic medications and pharmacy preferences to identify plans that offer improved insurance coverage. The software generated a print-out that can be shared with the patient. This printout contained information on the patient's current plan, with comparisons to the three lowest-cost Medicare Part D plan alternatives. Pharmacy staff informed patients that several plans existed, and the ones shown were associated with the lowest yearly patient expenditure. After explaining the components of benefit design (premium, copayment, deductible, etc.), the patient was informed on how to change plans. Patients were encouraged to make plan-switching decisions on personal devices but were allowed to use pharmacy computers to make same-day plan-switching decisions at the request of the patient.

### 2.3 Journey mapping platform: items, domains, and output

The online journey-mapping platform started technical development during the first quarter of 2017. The software team that built the platform regularly design and develop applications for projects where data sets are both collected and visually displayed via a web interface. The first journey-mapping beta project took place during the summer of 2017 and informed several changes related to the proprietary algorithm as well as updates to the user interface. As the beta program progressed, updates were implemented in collaboration with both the leadership team experienced with journey map deliverables and the development team familiar with valid approaches to data collection and visualization. The journey-mapping platform entered full production in November 2019 after multiple rounds of iterative updates from beta projects.

Survey questions were based on standardized items from the online journey mapping platform and tailored to fit the Medicare Part D plan selection experience. Survey items corresponded to predetermined journey mapping domains: *Discover, Search, Assess, Decide, Assist.* These domains focus on the entirety of the Medicare Part D selection experience. Using the standardized items, a study author with Medicare Part D consultation used an iterative process to refine items to reflect the Medicare Part D experience. In total, 7 demographic items, 30 Likert-type items, and 7 open-ended response items were initially included in the survey. Community pharmacy personnel providing the Medicare Part D consultation service reviewed the survey for initial validity and reliability. After revisions suggested by community pharmacy personnel, the survey was piloted with two Medicare Part D beneficiaries currently enrolled in a Medicare Part D plan but unaffiliated with the study pharmacy to identify issues with survey clarity. After piloting, modifications were made based on suggestions to improve readability. Journey mapping domains and example items from the specific journey-mapping platform are included in Appendix A.

Data from the returned paper surveys were entered into the online journey-mapping platform. The software used in this project includes algorithms and analytics that produce visual representations of the Medicare Part D journey for pharmacy service users and nonusers. The platform generated *Persona*, *Empathy*, and *Current Journey* outputs. The *Persona* outputs can be described as an avatar representing generalized characteristics of each survey group based on median responses. The *Empathy* outputs are a visual depiction of four emotion domains: *Think & Feel, Hear & See, Pain, Gains.* Multiple choice survey response items were mapped to each emotion domain and validated by the journey mapping platform development team. The *Empathy* output also procured an overall emotional experience score, quantifying the overall experience based on the range of emotions felt in the Medicare Part D plan selection process. Scores may range in values from 0 to 10, with larger values associated with better experiences. The *Current Journey* outputs provide a visual depiction of the comprehensive Medicare Part D plan selection process, progression through 5 decision-making domains: *Discover, Search, Assess, Decide, Assist.* The *Current Journey* outputs mapped *Good Experiences* and *Bad Experiences* on the Medicare Part D plan selection journey. These experiences are often denoted as *Touch Points* in journey mapping best practices.^17^^,^^18^ These moments provide an opportunity for service refinement (*Bad Experiences*) or depict moments of experience success (*Good Experiences*). During *Good Experience* moments, patients and customers may be further engaged with the service or experience and are more receptive to additional experiences.^17^^,^^18^

### 2.4 Study participants and recruitment

The pharmacy used purposeful convenience sampling to distribute a paper version of the journey mapping survey to patients between June and August 2019. Recent work developing patient journeys with healthcare services has considering data collection from as few as 8 participants sufficient to develop a patient persona.^20^, 21., 22 The online journey mapping platform requires 18 complete surveys for each condition to develop a corresponding persona and patient journey. The present study used two conditions: service users and nonusers. Patients were instructed that participating in the study would not impact their eligibility for future pharmacy services, their responses would be anonymized for the research team and remain confidential, and their responses would be used for service improvement. Survey participation was voluntary, with the community pharmacy only distributing surveys to patients who agreed to return the surveys upon completion. Patients were incentivized with $10 USD gift cards for successfully returning completed surveys to the community pharmacy. In total, thirty-six surveys were purposefully distributed between May and July 2019.

### 2.5 Analysis

Journey map outputs were reviewed and discussed with the platform developer. Scaled survey items were analyzed using Mann-Whitney *U* tests to identify significant differences in responses between service users and nonusers. Open-ended survey responses were coded by the research team using a qualitative content analysis approach.^23^ Open-ended responses were extracted from surveys into service user and nonuser categories. Two study authors independently coded patient responses, inductively generating descriptive and in-vivo codes. The authors met to discuss themes and representative quotes, generating major themes from descriptive and in-vivo codes. These themes were then assessed using a deductive approach, with themes cross-referenced to existing consumer and patient behavior theory. The COREQ guidelines were referenced to support reporting transparency, quality, and validity.^24^ To further support trustworthiness and authenticity for qualitative analysis; theoretical triangulation, thick contextual description, and fairness of qualitative data presentation were used.^25^

## 3 Calculation

Journey mapping was used as a practical application of the Design Thinking framework.^26^^,^^27^ The Design Thinking framework focuses on five sequential domains to collect and assess information pertinent to the patient experience: *Empathize, Define, Ideate, Prototype, Test.*^26^ Journey mapping is a tool commonly used to generate empathy maps and empathize with the patient experience, a process centered around gaining an understanding of the user and their needs to understand their perspective. Journey mapping, while less common with patient experience research, provides a practical application of the Design Thinking *Empathy* domain to better understand the patient and their needs. As a result, journey mapping may be a useful tool to focus efforts on defining problems patients may experience throughout their experience, exploring potential solutions to these problems, and pragmatically testing these solutions. The Design Thinking framework and practical applications of the framework's domains like journey mapping may be a viable option in future research for assessing patient-experience with pharmacy services and developing interventions and services using a patient-centered approach.

## 4 Results

In total, thirty-six journey map surveys were distributed, completed, and returned to the community pharmacy, for a response rate of 100%.

### 4.1 Persona outputs

**Fig. 1:**
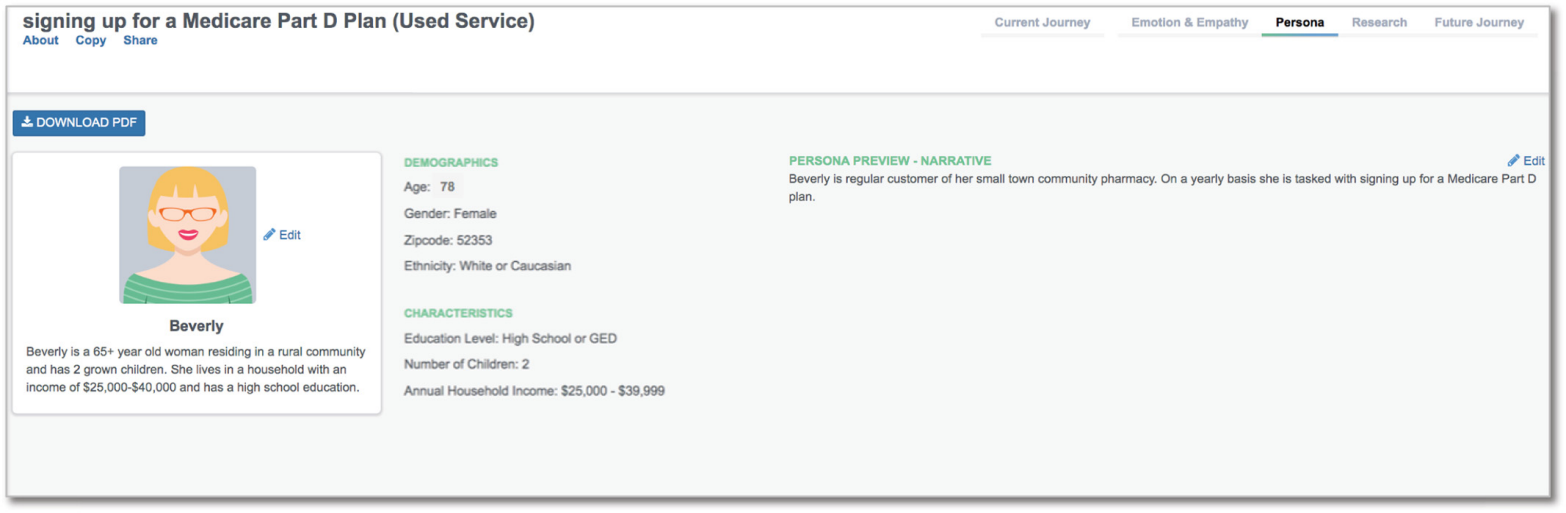
Persona for service usersPersona for service users

**Fig. 2:**
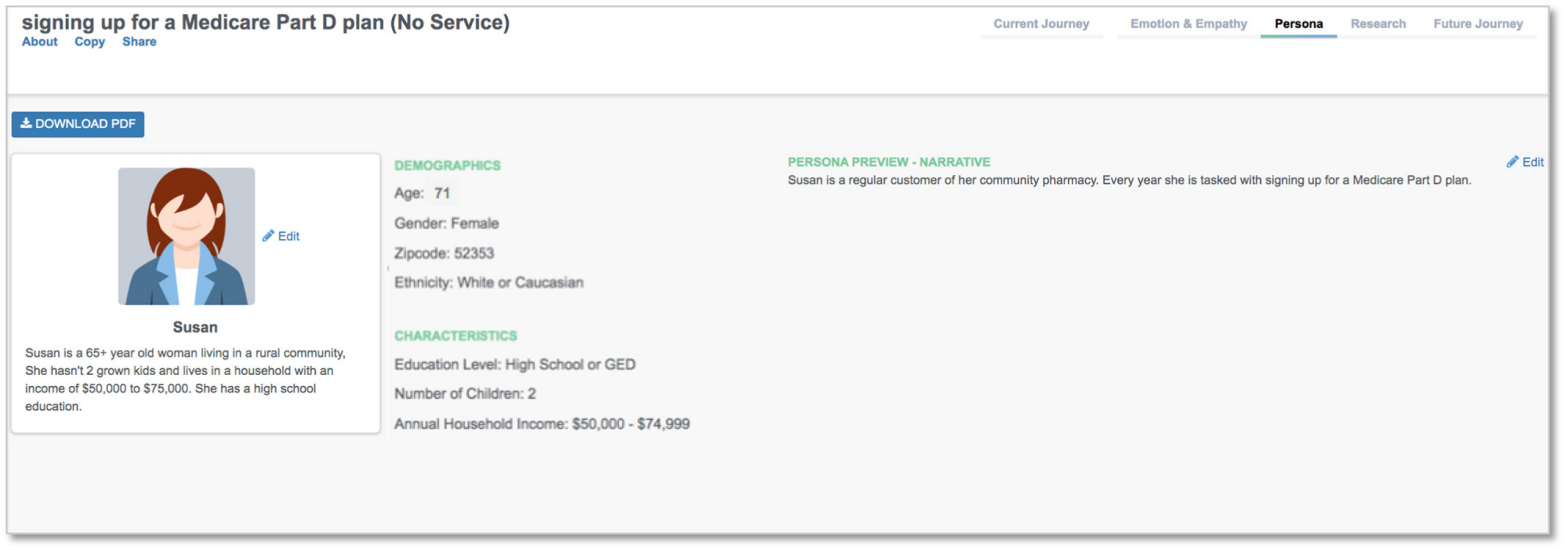
Persona for service nonusers

The two patient *Personas* were different in their household incomes. Individuals who did not use the service reported a median household income of $50,000–$74,999 compared to $25,000–$39,999 for those who used the service. Individuals who used the pharmacy consultation service were older than those who did not use the pharmacy consultation service. Journey mapping software outputs for *Persona* are included in Fig. 1, Fig. 2. A summary of basic patient demographic information can be found in Table 1.

**Table 1:** Service user and nonuser demographics.

Characteristics and demographics	Service users frequency (%)	Service nonusers frequency (%)
Age Mean Range	77.61 Min: 67 Max: 93	71.39 Min: 65 Max: 84
Gender Males Females	6 (33.3) 12 (66.7)	8 (44.4) 10 (55.6)
Education Level No High School High School or GED Some College Bachelor's Degree Master's Degree Advanced Graduate or PhD Not Sure/Prefer Not to Answer	0 (0) 7 (38.9) 4 (22.2) 4 (22.2) 1 (5.6) 1 (5.6) 1 (5.6)	0 (0) 8 (44.4) 5 (27.8) 2 (11.1) 0 (0) 3 (16.7) 0 (0)
Income Under $25,000 $25,000 to $39,999 $40,000 to $49,999 $50,000 to $74,999 $75,000 to $99,999 $100,000 +	4 (22.2) 3 (16.7) 3 (16.7) 2 (11.1) 2 (11.1) 4 (22.2)	1 (5.6) 1 (5.6) 4 (22.2) 9 (50) 2 (11.1) 1 (5.6)
Race White or Caucasian Other	18 (100) 0 (0)	18 (100) 0 (0)

### 4.2 Empathy point outputs

**Fig. 3:**
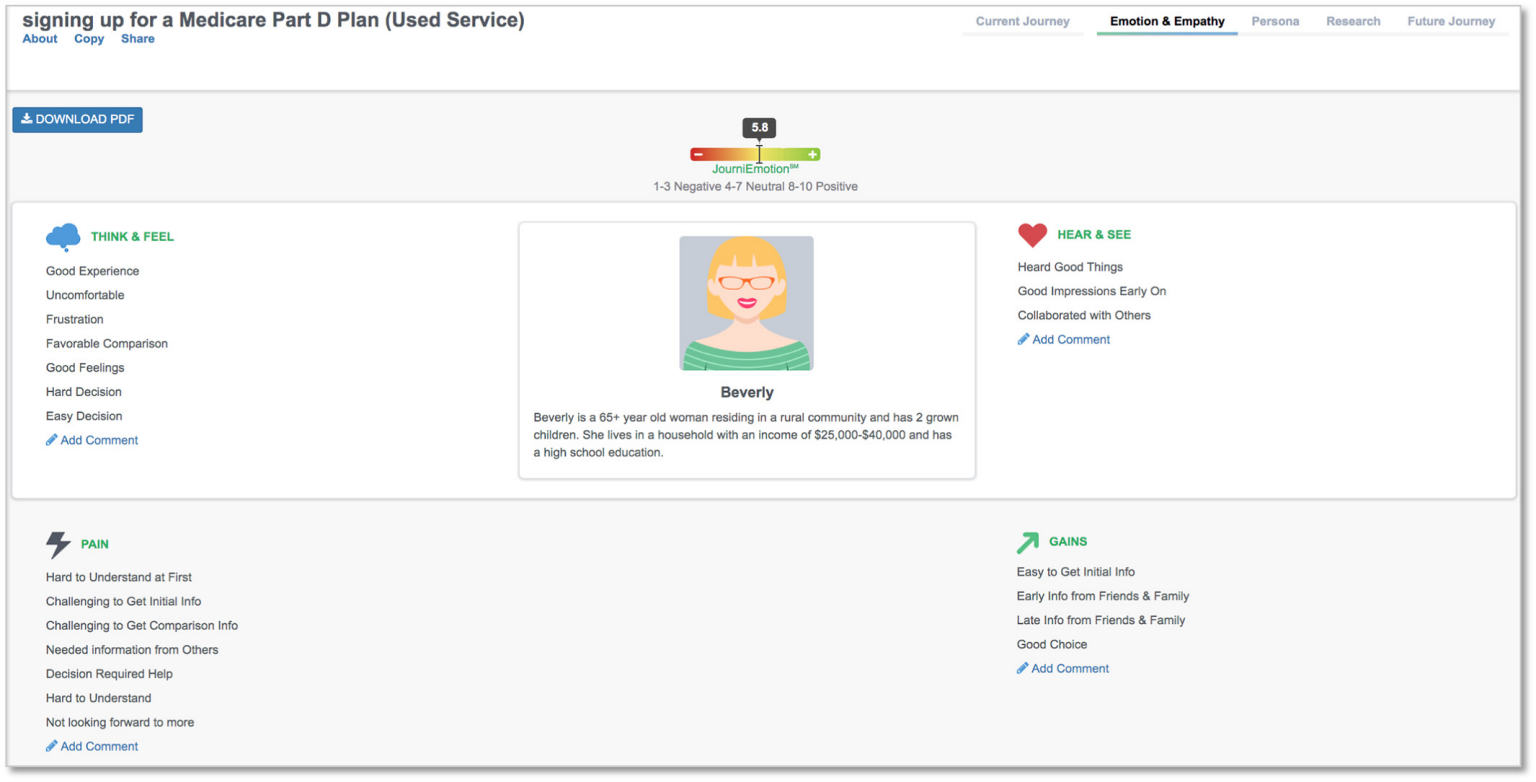
Emotion and Empathy output for service users

**Fig. 4:**
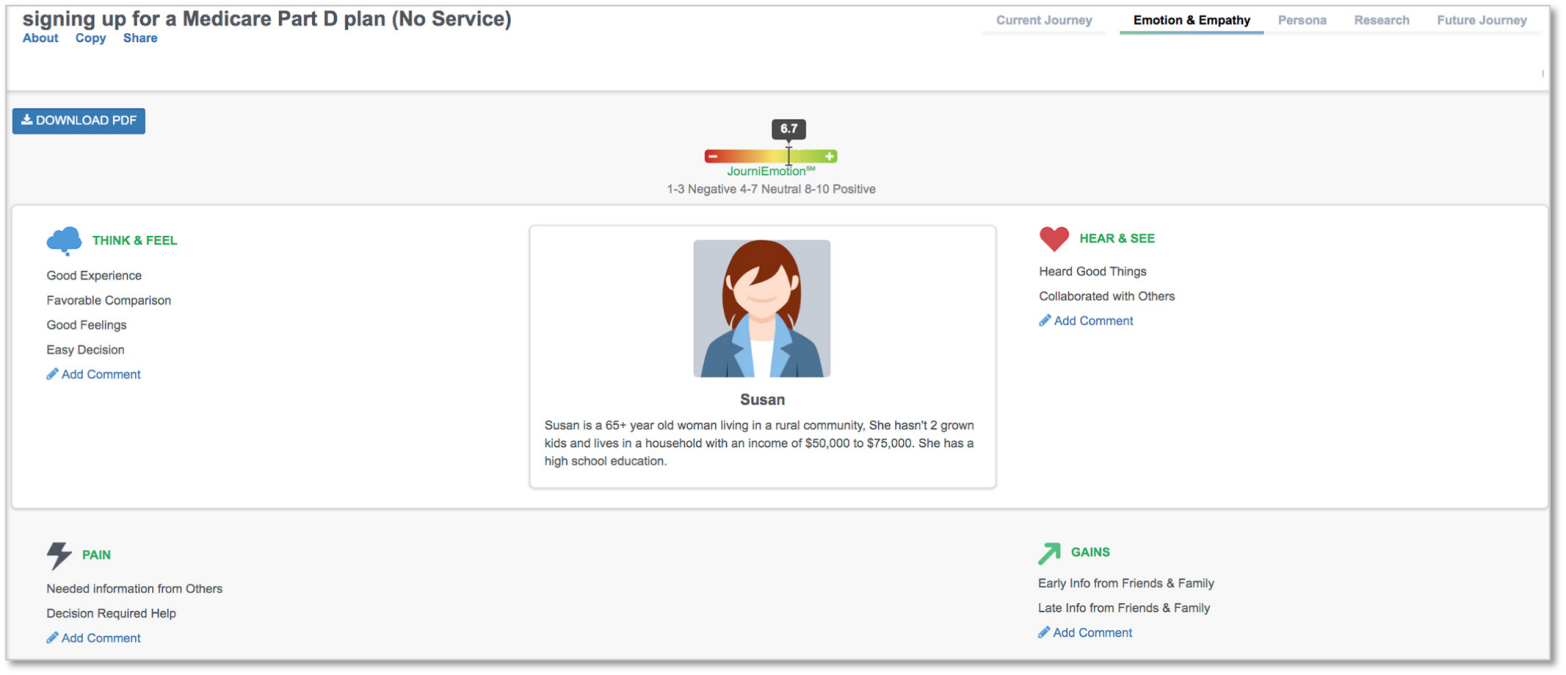
Emotion and Empathy output for service nonusers

Individuals who used the pharmacy service experienced more empathy points in each of the four empathy domains, with the largest visual difference between the groups in the *Think & Feel* category. Patients who used the service had a variety of experiences with the service, ranging from *Frustration* and *Uncomfortable* to *Good Feelings* and *Favorable Comparison**.*** Nonusers reported only positive experiences, including *Good Experience* and *Good Feelings.* Service users had more *Pain* and *Gain* responses than those who did not use the service. For individuals who used the pharmacy service, their overall emotional experience score was 5.8, while individuals who did not use the pharmacy service generated an overall emotional experience score of 6.7. *Empathy* outputs generated by the third-party journey mapping software are included in Fig. 3, Fig. 4.

### 4.3 Current journey outputs

**Fig. 5:**
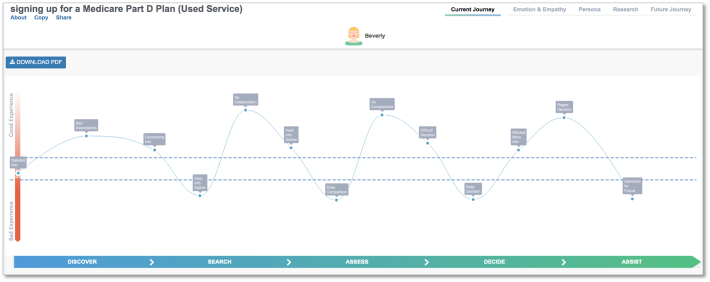
Current Journey for service users

**Fig. 6:**
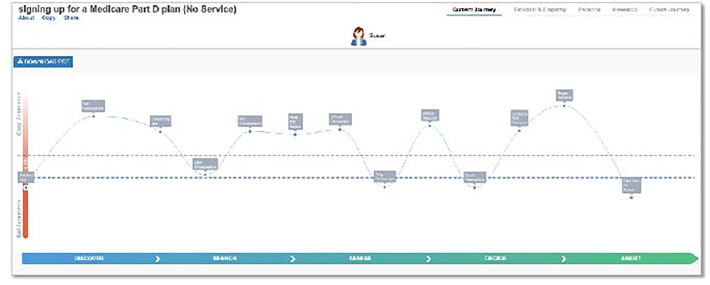
Current CJourney for service nonusers. Emotion Journey for serviice ce

From the *Current Journey* outputs, service users had more extreme *Good* and *Bad Experiences*. Service user journeys had more frequent and larger fluctuations in their experience compared to service nonusers. Areas with visual differences in the plan-selection journey appeared to involve collecting and assessing information related to Medicare Part D plans, the need for Medicare Part D assistance late in the decision-making process, and plan selection/decision-making processes. Despite different journeys, both groups appear to end up at similar places, with both groups reporting low optimism regarding their decision. Service users appeared to have more *Touch Points* during their Medicare Part D plan-selection journey based on the visual depiction of their *Current Journey* depicted in Fig. 5, Fig. 6.

### 4.4 Survey analysis

Mann-Whitney *U* tests showed that five survey items had responses with statistically significant differences between service users and nonusers. Most notably, individuals who used the service had a greater appreciation for the features or benefit designs of other plans (mean rank of 23.25 for service users compared to 13.75 for nonusers, *p*-value <0.05) and felt they had more opportunities to compare Medicare Part D plans (mean rank of 22.56 for service users compared to 13.69 for nonusers, p-value <0.05). General item categories and outputs are included in Table 2.

**Table 2:** Comparison of selected journey mapping prompts for users and nonusers of the consultation service.⁎

Item	Group (N)	Mean Rank	*sig*
Opportunity to Collect and Assess Information	User (18)	22.75	0.014
	Nonusers (18)	14.25	
Required Late-process Assistance	UserUser (17)⁎⁎	22.56	0.009
	Nonuser(18)	13.69	
Opportunity to Make Plan Comparisons	UserUser (18)	22.56	0.020
	Nonuser (18)	14.44	
Difficulty with Plan Selection	User (18)	22.44	0.024
	Nonuser (18)	14.56	
Awareness of Other Plan Features	UserU (18)	23.25	0.006
	Nonuser (18)	13.75	

⁎ Mann-Whitney U test with 2-tailed level of significance *p* = 0.05. Mean Rank values, higher values suggest increased awareness of opportunity or component with the Medicare Part D plan selection experience.

⁎⁎ *n* = 17 due to missing response.

### 4.5 Open-ended response analysis

After thematic analysis, the authors agreed on two major themes that describe patient plan-selection experiences: “complexity and uncertainty in the decision-making process” and “the value of trust in the plan selection process.” Representative quotes from service nonusers [NU] and service users [U] may be found in Appendix B.

#### 4.5.1 Complexity in the decision-making process and information uncertainty

Patients who did not use the pharmacy service appreciated how challenging and complex the plan selection process could be. These individuals expressed varying degrees of frustration with the plan-selection process but often used non-pharmacy resources to help make their plan decision. In some instances, the individuals who did not use a pharmacy service seemed more comfortable deciding on their own and appeared more aware of the various components of benefit design and different components of their specific insurance plan, often engaging in independent research and plan comparison. Individuals who did not use the service used a wide variety of information sources to make their plan decisions, including family, friends, and insurance agents. When individuals sought help, they often let the individual make their plan selection for them.

*You must look at every detail to get the best deal for you-company, pharmacies, medications. [NU9].*

*It is hard to decide if you want a higher deductible or cheaper drug cost. [NU1].*

*Just went to Farm Bureau and let them decide. [NU14].*

*Went with AARP suggestions, overwhelmed with doing it on my own. [NU2].*

For individuals who did use the pharmacy service, they appeared more aware of the number of plans that were available to them. While some patients appreciated the opportunity to compare plans, many found the plan-comparison process to be frustrating, specifically covering details such as medication coverage. In one instance, the comparison process drove an individual to select a plan from a previous year. While some patients felt the pharmacy service presented options in an understandable form, others reported there were aspects of plan information they did not understand.

*There are so many options for plans [U4].*

*Least enjoyable: comparing what they did or did not cover. So much paperwork to go through [U7].*

*Comparison left me frustrated, went back to my original plan because my agent can help me. [U12].*

*The most enjoyable part was having my options presented to me and explained in an understandable form. [P10].*

#### 4.5.2 The value of trust

Both groups of individuals had a challenging time trusting the Medicare Part D plan information they received but were aware that plan-switching may be beneficial every year. Individuals who did not use the pharmacy Medicare Part D consultation service were often still uncertain of their plan decision but seemed to have high levels of trust with the individual that assisted them with their plan decision and/or the information they acquired. Ultimately, service nonusers readily accepted the recommendations provided by trusted agents.

*Some uncertainty at first, but confidence because my husband talked to his health insurance agent. [NU12].*

*I took the advice of trusted agents. [NU13].*

For individuals using the pharmacy service, trust was more challenging to establish. Patients using the pharmacy service had a difficult time trusting the pharmacy staff member providing the consultation and the information they received. In some instances, patients felt like the pharmacy staff member was trying to “sell them” a specific plan. Ultimately, service users were often uncertain if they had selected the best plan.

*I needed to trust the person selling it. Hopefully she was honest and not just working to make a sale. [U6].*

*Least enjoyable was trusting the decision I made was best for me [U14].*

*Still not confident I have the best plan for me!! [U14].*

## 5 Discussion

The journey mapping process highlighted important differences in the plan-selection experience for service users and nonusers. These differences in experience occurred over the entire service process and were highlighted by differences for users and nonusers in the two personas and the open-ended comments.

Commonalities between pharmacy service users and nonusers were that all individuals found the Medicare Part D plan-selection process to be challenging, highlighting that collecting information and assessing it for trustworthiness was difficult, especially in such a technical domain. Both groups reported depending on additional assistance with their Medicare Part D plan selection, but their access to, and use of trusted experts to help with decision-making varied. Despite the differences in information acquisition and plan comparison, both groups still had doubts about their ultimate plan decision, suggesting a target for improvement.

One important difference between pharmacy service users and nonusers was their average income level. Older individuals with lower fixed incomes appear to have sought additional support from their community pharmacy more often than younger individuals with more financial resources. People with higher incomes may already have access to services like a financial planner or an insurance agent who may be providing advice. Less well-off or older adults may have less access to these resources.

Another difference was that individuals who used the Medicare Part D consultation service reported more opportunities for plan comparison and greater awareness of other plan features than those who did not use the pharmacy service. This points to the pharmacy conferring their unique expertise – but perhaps doing so at a level that exceeds the patient's desire for and capacity to use complex information. Consistent with other studies, this level of information overload seems to have led to patient's having a more challenging plan-selection experience.^28^^,^^29^ Pharmacists should consider how presenting information in this way could be incompatible with a patient's health insurance numeracy and literacy, which research suggests may be challenging for older patients.^30^^,^^31^ Further, since patients who used the pharmacy service often deferred to the judgment of their pharmacist or technician based on trust, streamlining the information provided and affectively reassuring the patient as a trusted professional may be a better approach than trying to gain trust through providing comprehensive data. Describing how the pharmacist came to the recommendation may yield more trust than pages of plan comparisons.

Individuals who did not use the service, on the other hand, appeared to already have a better baseline understanding Medicare Part D insurance and the plan-selection process, often highlighting the importance of understanding specific insurance jargon (i.e. deductible) in their open-ended comments and choosing plans based on this understanding. Individuals who did not use the community pharmacy service may not have been as overwhelmed with information if their agent offered a narrower recommendation, but still left their decision up to an individual they had previously trusted with difficult financial decisions. While, it is difficult to disentangle what effect might be due to education and income and what effect is due to the pharmacy service itself, the data point to targets for service improvement focusing on patient-centered information and facilitating trust within the encounter.

### 5.1 Trust in older populations

With patients making their Medicare Part D plan decision based on the extent to which they could trust the information presented by the pharmacy team, it is important to explore how trust influences older individuals and their decision-making processes.^32^^,^^33^ While individuals become increasingly trustworthy as they age,^34^ information emphasizing financial implications tends to decrease levels of trust in older populations.^35^ In addition to the effects of cost-related messaging, trust may also be influenced by the level of detail and the amount of information presented to older populations.^32^, ^33^, ^34^, ^35^, ^36^ Older individuals prefer benefit information (information about the general improvements they may experience as a result of a decision) compared to attribute information (specific information to inform a decision such as plan attributes and benefit design).^34^^,^^35^ Based on the pharmacy service description, it appears that the information provided by staff emphasized specific plan attributes and cost-related information like premiums, copayments, deductibles, and medication costs. While the pharmacist and pharmacy technician may have presented information they felt the patient needed to make an informed decision, the information unintentionally decreased patient trust. To encourage patients to use Medicare Part D consultation in multiple years and feel comfortable using the information obtained during these services, community pharmacies would stand to benefit from addressing how information influences service heuristics like trust.^37^^,^^38^

### 5.2 Understanding trust using the elaboration likelihood model (ELM)

The *Elaboration Likelihood Model* (ELM) may be a useful model for conceptualizing the relationship between patient preferences for information and trust during challenging decision-making experiences like selecting a Medicare Part D plan.^39^^,^^40^ The ELM posits that individuals use a dual-process approach when presented with persuasive information, which will be used when making decisions. The ELM focuses on an individual's ability to *elaborate*, or the ability to perform issue-relevant thinking. The extent an individual elaborates influences the types of persuasive messaging and information they may be receptive to, specifically central and peripheral persuasive messaging. Central routes to persuasion often are useful when elaboration is high. Individuals who engage in high levels of issue-relevant thinking undergo careful processing of information and message, ultimately using the information to make an informed decision. Peripheral persuasion is less direct, where individuals use simple decision values like communicator credibility or trustworthiness to make their decision. Elaboration for individuals influenced by peripheral persuasion messages is low, with issue-relevant thinking minimally involved in persuasion and the subsequent decision-making process.

In the context of the Medicare Part D consultation service, patients were exposed to a multitude of plans with an emphasis on specific benefit designs. Patients were required to perform high levels of elaboration, processing large amounts of complex information and use it to make informed Part D plan selections. By providing patients with high-level detailed plan information, the pharmacy assumed that patients were willing and able to elaborate. When faced with plan comparison opportunities via the community pharmacy service, patients were consistently frustrated and reported negative experiences, which may be attributed to moments requiring high levels of elaboration such as processing information and comparing plans to make a plan-switching decision. Patients who did not use the service were infrequently required to elaborate, as patients who did not use the pharmacy service were presented with considerably less information and were infrequently required to compare plans, which resulted in a better experiences potentially due to lower elaboration requirements. When patients who used the pharmacy service were unable to elaborate, they defaulted to heuristics, like the trustworthiness of the pharmacy team, to make their decision. Given that the information presented to patients emphasized cost-related features of specific plan information, patients may have defaulted to a trust heuristic which may have worsened the plan-selection experience. Individuals who were able to elaborate, or unable but not required to elaborate, may have had more positive experiences as information and persuasive messaging were aligned with patient ability and preference.

### 5.3 Future research

Patient-centered design and consumer behavior research methodologies can be used to refine community pharmacy interventions and the associated impacts of these refinements on patient behavior and outcomes. While we used a journey mapping software platform, more traditional ethnography methodology and participatory approaches can be used to evaluate the patient experience and facilitate a better understanding of the patient decision-making process. To corroborate findings, additional methodologies should be employed, such as patient observations, interviews, and focus groups. There may be additional benefit in prototyping other interaction designs for community pharmacy services, specifically focusing on Medicare Part D consultations, to compare across patient-pharmacist interaction evaluations. For example, how should messages about plan cost be framed so the patient does not feel like the pharmacist has a financial conflict of interest? Research should be conducted to explore differences in patient perceptions of services based upon the person providing the service: pharmacist, pharmacy intern, or pharmacy technician. As journey mapping does not formally test patient preferences and needs related to an experience, more work is needed to formally test and evaluate patient preferences for Medicare Part D consultation service delivery, including the types of information patients prefer, how users would like information presented, and the level of information complexity that users need to make informed Medicare Part D plan decisions.

### 5.4 Limitations

There are several study limitations to consider. Data were collected from a single, rural community pharmacy located in rural Midwest USA. Other pharmacies may have alternative ways of delivering Medicare Part D consultation services and more diverse patient populations may have different experiences. The community pharmacy distributed and collected surveys that may have contributed to social desirability bias, however, patients who used the service reported worse Medicare Part D experiences than those who did not. Both a pharmacist and pharmacy technician delivered the service, and as a result, experiences may have differed based on the service provider. Further, there may have been variations in the patient experience based on the method of service delivery (telephonic vs. in-person). These service variations are likely to contribute to variations in the patient experience but were unable to be linked to specific survey responses. As such, patient preference for delivery method and service provider should be explored in future studies.

## 6 Conclusion

Using a journey mapping process that included qualitative and quantitative data collection and analysis depicted the entirety of a patient experience, both good and bad, with selecting a Medicare Part D plan, with or without using a pharmacy-provided Part D plan selection service. Quantitative data triangulated these results and identified key differences in experiences and demographics between community pharmacy Medicare Part D consultation service users and nonusers. Qualitative data provided context and themes informing theoretical applications and explanations associated with the patient experience. By integrating these methodologies, consumer trust literature, and ELM, we can identify clear areas of pharmacy service improvement with recommendations for patient-centered refinement informed by existing theory. Based on these findings, community pharmacies offering Medicare Part D consultations would benefit from redesigning interventions to focus on the way information is delivered to patients, what information is provided, and how they can instill trust in the patients they serve.

## Funding

This research did not receive any specific grant from funding agencies in the public, commercial, or not-for-profit sectors.

## Declaration of Competing Interest

The authors declare that they have no known competing financial interests or personal relationships that could have appeared to influence the work reported in this paper.

## Figures and Tables

**Table:** 

Journey map survey domains and open-ended response category	Example items
Discover	When I first heard about signing up for a Medicare Part D plan or initially learned about it, I would describe discovering this information as a positive experience. Before I learned more detailed information about selecting a Medicare Part D plan, I initially had positive thoughts or feelings about it.
Search	I did not consult others when seeking to learn more about selecting a Medicare Part D plan. While gathering information about selecting a Medicare Part D plan, I felt uncomfortable, personally concerned, or somewhat worried.
Assess	Understanding what each Medicare Part D plan offered, as compared to other plans, was clear and easy. When it came time to select a Medicare Part D plan, it was difficult to collect the right information for a valid assessment of my options.
Decide	When it came time to select a Medicare Part D plan, I found it difficult to make a decision. I felt good about what my Medicare Part d plan selection compared to alternative choices.
Assist	I look forward to the experience of selecting a Medicare Part D plan in the future. I wish I had more and/or better information before selecting a Medicare Part D plan.
Open-ended Response Items	Describe the last time you selected a Medicare Part D plan. Briefly describe what was most enjoyable or least enjoyable about your process of finding information about selecting a Medicare Part D plan. What other comments do you have about your process in selecting a Medicare Part D plan?

**Table:** 

Theme	Additional reponses (service status & patient #)
Complexity in the decision-making process and Information Uncertainty	**Service Nonusers** *My agent. [NU4]* *Agent was very helpful, really wasn't very difficult. [NU7]* *I discussed options with friends, relatives, the insurance agent was knowledgeable and answered my questions. [NU7]* *When I retired-took employers retirement plan. [NU10]* *Used an agent to make the selection and continue to use them for questions. They have been great! [NU15]* *My agent was great at explaining Part D. [NU4]* *I liked computer access to compare “D” plans. [NU6]* *Comparing alternative part D plans to find best option. [NU6]* *Length of time we were on the phone going over all of the details and medications. [NU8]* *Insurance plans are a frustrating experience for me. I choose the best plan for my situation. [NU16]* *Confusing, frustrating, Glad to have help through my husband's company. [NU8]* *“Easy” but frustrating to have to compare plans/pharmacies* etc. *[NU6]* *Finding information in different forms on different formats, why not have universal form [NU2]* *Selection done with a graph of meds and who offers what. [NU2]* *I rely on my medical insurance provider to help me select the best plan [NU1]* *Found an agent out of Cedar Rapids that offers several plans-not just one-see him at the end of year. [NU9]* *I used the internet-ignored any that could afford long TV ads-looked for lower cost since I take inexpensive medication and am in good health. [NU12]* **Service Users** *I am looking forward to my pharmacy to help me select the best option for 2020. [U7]* *Horrible. I am happy with the information given to me by the pharmacy but did feel horrible about it before asking for help. [U5]* *The enjoyable part was having [redacted] help in choosing because they had direct knowledge of our drug usage. The least enjoyable was not totally being sure of our decision when we picked it ourselves. [U15]* *Too many options [U3]* *Just glad it is over with [U4]* *It was ok. Kind of scary [U15]* *The pharmacy associate was very good to look at the different Part D programs and lead me in the right direction! [U9]* *Not knowing who to talk to [U6]* *I needed information and was able to find it by the help of my pharmacists [U4]* *Did not understand all aspects [U11]*
Trust	**Service Nonusers** *You need professional help that can sort everything out! [NU1]* *Making a positive decision. [NU17]* *Having to change providers almost yearly. Having different plans for each [of us] [NU6]* *We listened to the advice of our insurance agent. [NU3]* *Talked to family members about their plans. Talked to agent. Made my decision quickly. [NU18]* *Seems like I have to change every year, because they [the plans] change [NU1]* *Careful consideration of comparative plans [NU6]* *Having help so you don't feel like you're alone and possibly making a wrong decision. [NU9]* *We left it up to the agent. [NU3]* *My Farm Bureau agent was great, I did not look at other options. [NU12]* *Have no choice, just accept. [NU17]* ***Service Users*** *That what I was told would happen with my choice was true! [U9]* *It was a relief to know that it was taken care of and by knowledgeable help [U10]* *Still not confident I have the best plan for me!! [U14]* *Hope you make the right move, do the right thing. (U16]* *Before [redacted] pharmacy helped us, we relied on and trusted friends and professionals for assistance. [U16]* *I had to take the advice of the person selling it [U6]* *My wife and I made the decision together [U13]* *At first it was like a leap of faith, but we realized we could change it in a year if we were not satisfied. Almost like trial and error when we first went on Part D [U16]*
